# The pros and cons of remote work in relation to bullying, loneliness and work engagement: A representative study among Norwegian workers during COVID-19

**DOI:** 10.3389/fpsyg.2022.1016368

**Published:** 2022-10-25

**Authors:** Veronica Bollestad, Jon-Sander Amland, Espen Olsen

**Affiliations:** Department of Innovation, Management and Marketing, Business School, University of Stavanger, Stavanger, Norway

**Keywords:** remote work, loneliness, work engagement, bullying, COVID-19, psychological health, well being

## Abstract

Remote work became the new normal during COVID-19 as a response to restrictions imposed by governments across the globe. Therefore, remote work’s impact on employee outcomes, well-being, and psychological health has become a serious concern. However, the knowledge about the mechanisms and outcomes of remote work is still limited. In this study, we expect remote work to be negatively related to bullying and assume that bullying will mediate remote work’s impact on work engagement and loneliness. To test our hypothetical model, we applied a cross-sectional design using data from a large representative sample of 1,511 Norwegian workers. The data were collected in September 2021 during a period of COVID-19 restrictions in Norway. The results confirmed our hypotheses: remote work was positively related to loneliness and work engagement but negatively related to bullying. Further, bullying was positively related to loneliness and negatively related to work engagement. Moreover, bullying was also found to play a partial mediating role, supporting our hypothesis. This study suggests that remote work is related to both positive and negative mechanisms in the workplace. Remote work can potentially reduce bullying and have a protective function in preventing bullying. However, since remote work has positive relations with both loneliness and work engagement, this study illustrates that organizations should be cautious and perhaps consider a moderate level of remote work. Hence, the results have several implications for HR policies and management.

## Introduction

The COVID-19 outbreak caused a rapid shift into full-time remote work for millions of employees all over the globe (Contreras et al., 2020; Yang et al., 2022). Without any preparations, remote work became the new normal (Schur et al., 2020), even in positions we previously assumed had to be done on-site (Savić, 2020; Sytch and Greer, 2020). Remote work represents a fundamental shift in organizational work design (Wang et al., 2021) and completely changes physical and psychological interactions, possibilities, and relationships (Konradt et al., 2003; Gajendran, 2007; Contreras et al., 2020; Schur et al., 2020; Yang et al., 2022). This shift in work design makes it important to investigate remote work’s effect on important mechanisms at the workplace.

The extent to which employees were able to adjust to remote work is crucial for the individual- and organizational outcomes, such as mental health, well-being, and work engagement (van Zoonen et al., 2021). As a result, research on remote work in the aftermath of COVID-19 is increasing, and remote work has become a topic of great scholarly interest (e.g., Brynjolfsson et al., 2020; Ozimek, 2020; Popovici, 2020; Ferreira et al., 2021; Wang et al., 2021; Pokojski et al., 2022; Yang et al., 2022). However, there is still a lack of research on remote work’s potential effect on workplace bullying. Bullying is claimed to be the most severe social stressor in the workplace, and in-person interactions are an important driver of bullying (Bacher-Hicks et al., 2022). Therefore, it is important to investigate bullying in the context of remote work, where targets and bullies are physically separated. This study aims to fill this gap by developing a theoretical model that explores the mediating role of bullying in relation to remote work and its effect on loneliness and work engagement. It investigates the effects of remote work on Norwegian employees almost 2 years into the pandemic. Furthermore, it seeks to address the gap in the existing literature.

This study assumes that remote work will substantially influence social interactions at work, thereby reducing perceptions of bullying and influencing workers’ perceptions of loneliness and work engagement. We seek to understand the relationships between remote work, bullying, loneliness, and work engagement, and seek to gain information about these unexplored, yet important issues affected by remote work. Based on theory, we will develop and test a theoretical model in a representative sample of workers in Norway. This study will provide new insights and knowledge about the versatile influence of remote work in the workplace.

## Theoretical background and research hypotheses

### Remote work and loneliness

Loneliness is an important factor in organizational contexts. For example, employee loneliness is negatively related to well-being, creative performance, organizational citizenship behavior, job satisfaction, and job performance (Wright, 2005; Erdil and Ertosun, 2011; Ozcelik and Barsade, 2018; Firoz and Chaudhary, 2021). Loneliness is a psychological state that occurs when there is a discrepancy between the interpersonal relationships one wishes to have and the relationships one has (Peplau and Perlman, 1982). Those who experience difficulties establishing and maintaining interpersonal relationships struggle to address their need for belonging and are more likely to experience loneliness (Baumeister and Leary, 1995; Cacioppo et al., 2000). Loneliness is experienced by adults of all ages (Ozcelik and Barsade, 2018), and influences how people feel and behave towards others and how others feel and behave towards them (Heinrich and Gullone, 2006; Cacioppo and Hawkley, 2009). Even though loneliness may be experienced differently based on personality traits (Buecker et al., 2020), it is particularly important for organizations to address loneliness, as positive employee interactions play a significant role in employees’ motivation and satisfaction at work (Dutton and Heaphy, 2003; Wang and Brower, 2019).

It was only recently that studies began investigating the relationship between remote work and loneliness. Remote work completely changes social interactions, social possibilities, and social relationships (Konradt et al., 2003; Contreras et al., 2020; Schur et al., 2020; Yang et al., 2022). Employees working remotely may feel more lonely as they have fewer in-person interactions, are more exposed to social isolation, and lose the opportunity to meet friends and colleagues (Hwang et al., 2020; Wang et al., 2021; Buecker and Horstmann, 2022). Further, a study by Carillo et al. (2021) points out that lack of contact and informal relationships with colleagues and lack of feedback from managers and organizations are major problems for remote work. The lack of contact and informal relationships makes it difficult to maintain interpersonal relationships digitally. Therefore, we propose the following hypothesis (H):

*H1*: Remote work is positively associated with loneliness.

### Remote work and work engagement

Organizations must facilitate and inspire full engagement for their employees. Work engagement can truly make a difference for employees and may result in competitive advantages, such as increased job performance (Bakker and Demerouti, 2008). Work engagement is defined as “a positive, fulfilling, work-related state of mind that is characterized by vigor, dedication, and absorption” (Bakker et al., 2011, p. 5). Engagement is predicted by typical job resources (Bakker and Demerouti, 2007). For example, social support from colleagues, performance feedback, skill variety, and autonomy (Bakker and Demerouti, 2008). Engagement is a motivational concept, increasing personal growth, development, and performance. Overall, producing positive outcomes at an individual and organizational level (Bakker and Leiter, 2010).

Despite the increased prevalence of remote work, its direct impact on work engagement remains relatively unexplored. For example, new research on work engagement during COVID-19 explored predictors, gender differences, and possible relationships with work engagement (Gopalan et al., 2021; Koekemoer et al., 2021; Ojo et al., 2021; Rožman et al., 2021), but have left the direct effects of remote work on work engagement unspecified. Palumbo (2020) argues that remote work positively affects work engagement, since remote work empowers employees to harmonize work and family-related commitments and increases work-life balance. Some studies have also found that remote work increases productivity, which is highly correlated with work engagement (Ozimek, 2020; Toscano and Zappalà, 2021). Furthermore, remote work reduces commuting time, unnecessary meetings, and distractions in the office (Ozimek, 2020), ultimately giving employees more time to engage in their work. It can be argued that remote work reduces work engagement through work/life balance as it may cause more distractions (e.g., shopping, hanging with friends, housework) than being physically at the workplace. However, based on the literature it seems reasonable to assume that remote work increases work engagement. We thus propose the following hypothesis (H).

*H2*: Remote work is positively associated with work engagement.

### Remote work and bullying

Workplace bullying is defined as “repeating and enduring aggressive behaviors that are intended to be hostile and/or perceived as hostile by the recipient” (Einarsen, 1999, p. 18). Long-term exposure to bullying is more damaging for the recipients than all other kinds of work-related stress put together, as long-lasting bullying may cause severe psychosomatic and psychological problems for the target (Hauge et al., 2010; Mikkelsen et al., 2020). Moreover, employees exposed to bullying show lower levels of satisfaction and commitment at work, and their desire to remain with an employer and their willingness to be present at work decreases (McMahon, 2000). Bullying is found to be strongly associated with in-person interactions (Bacher-Hicks et al., 2022). Knowing this, workplace bullying represents a critical area of research. Especially in times of extensive use of remote work, where in-person interactions between employees are removed.

As a response to being bullied, victims could see it as a psychological necessity to either quit the job or take sick leave (O’Donnell et al., 2010). This way of separating themselves from perpetrators and leaving the situation is found to be the most effective coping strategy for bullied victims (Aquino and Thau, 2009). However, in terms of salary and commitments to other obligations, attendance at work is necessary and unavoidable (Hauge et al., 2010). Previous studies on remote work emphasize the positive aspects of employees choosing to work from home to avoid certain aspects of organizational life, such as bullying and other negative social acts (Mirchandani, 1998; Collins et al., 2016). Furthermore, a study by Karatuna (2015) found the physical separation of perpetrators and targets helped to de-escalate conflicts and end the bullying. In the case of remote work, the separation of perpetrators and targets happens naturally, since it allows employees to conduct work outside the traditional office. Furthermore, remote work could mitigate feelings of social exclusion (e.g., not being included in small talk, meetings, or lunches), as these social interactions are less visible or even eliminated when working remotely.

Based on this, our study assumes that remote work will have a positive impact on workplace bullying. Remote work removes in-person contact between employees and physically separates perpetrators and victims. Thus, the following hypothesis is proposed (H):

*H3*: Remote work is negatively related to bullying.

### Bullying, loneliness, and work engagement

Being a target of bullying has negative consequences on health-related and job-related outcomes (Trépanier, 2014; Khalid and Ishaq, 2015; Gupta et al., 2017). Furthermore, workplace bullying can severely affect organizational productivity and represents a significant source of social stress at work (McMahon, 2000; Vartia-Väänänen, 2003; Bano and Malik, 2013).

First, workplace bullying negatively affects the basic human need for belonging (Baumeister et al., 2007). Moreover, the experience of being bullied affects one’s ability to feel socially included in the organization (Fattori et al., 2015), loneliness and social isolation are consequences of bullying (Hogh et al., 2012; Campbell, 2013). According to Wright et al. (2006), loneliness is strongly related to the desired quality of interpersonal relationships. Therefore, the lack of high-quality relationships in work environments due to bullying could cause loneliness. Furthermore, loneliness caused by bullying is damaging to the affected person, causing stress, anxiety, and other health problems (Lewis and Orford, 2005; Green, 2021).

Second, workplace bullying has a negative impact on work engagement (Trépanier, 2014; Park and Ono, 2017; Goodboy et al., 2020). Victims of bullying report problems concentrating, self-doubt, decreased job satisfaction, and decreased productivity (Hallberg and Strandmark, 2006; Yıldırım, 2009; O’Donnell et al., 2010; Trépanier, 2014; Mikkelsen et al., 2020). Furthermore, several studies report that bullied victims have higher absenteeism, lower dedication, and lower commitment to work, all of which are negatively related to work engagement (McMahon, 2000; Yıldırım, 2009; Trépanier, 2014). Hence, being a victim of bullying is damaging to the affected person and has a direct effect on performance and psychological health. Thus, we hypothesize as follows (H):

*H4*: Bullying is positively related to loneliness.

*H5*: Bullying is negatively related to work engagement.

### Bullying as a mediator

From the theoretical framework presented above, we predict that remote work is negatively associated with bullying. Furthermore, we propose that remote work is negatively related to loneliness and positively related to work engagement and that bullying is negatively associated with work engagement and positively related to loneliness. This study will explore how bullying might mediate remote work’s influence on work engagement and loneliness. We seek to investigate whether remote work provides a protective mechanism against bullying.

A study by Olsen et al. (2017) revealed that bullying mediates the influence of job resources and demands on job performance, job satisfaction, and work ability. Hence, bullying has been shown to mediate the association between social interactions and outcomes at work. Our study hypothesizes that the perception of being bullied is reduced by working from home and therefore assumes that bullying will mediate the impact of remote work on loneliness and work engagement. First, we expect that the experience of workplace bullying will have significant consequences for work engagement since perceptions of being bullied are stressful experiences with negative effects on vigor (high mental energy), dedication (high involvement in work), and absorption (high concentration and engrossment in work; Goodboy et al., 2020). Second, bullying has severe negative consequences on the social environment (Einarsen et al., 1994). Since being bullied does not reflect the desired quality of interpersonal relationships, it has positive associations with feelings of loneliness (Wright et al., 2006).

Moreover, bullying is an interpersonal behavior intentionally aimed at causing harm to another employee (Bowling and Beehr, 2006). Since remote work reduces interpersonal contact, it is reasonable to believe that remote work will be negatively associated with bullying. This is reflected in a study by Golden and Gajendran (2019), which found that employees who experienced low levels of social support at work were positively associated with remote work. Furthermore, as low social support is positively associated with being bullied (Evans et al., 2014), we assume remote work will influence social interactions in the workplace, and that bullying will mediate the influence of remote work on loneliness and work engagement. As the above theoretical framework proposes, the physical and psychological separation induced by remote work could have positive outcomes. Thus, we propose the following hypothesis (H):

*H6*: Bullying will mediate remote work’s associations with (a) loneliness and (b) work engagement.

### Research model

**Figure 1:**
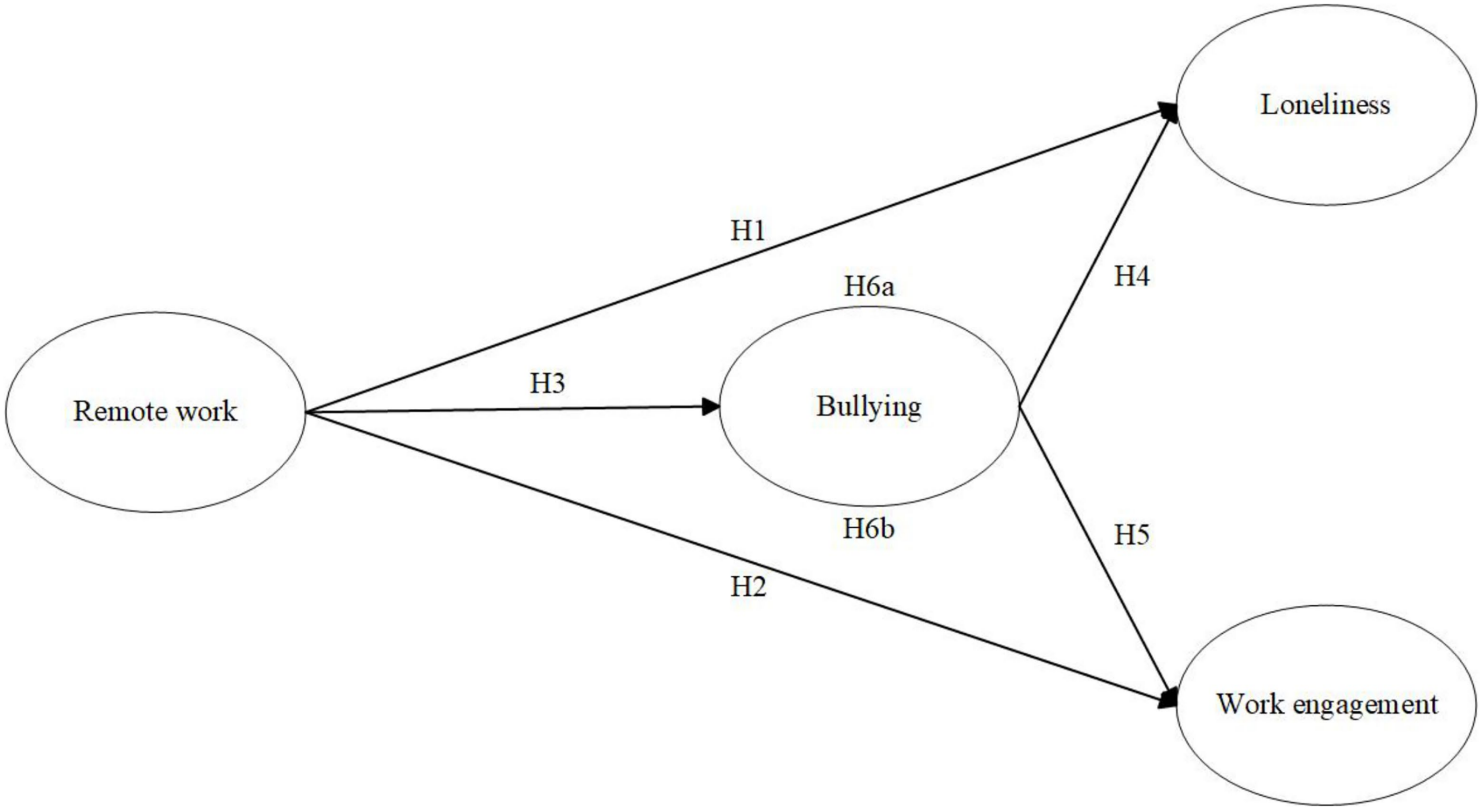
The research model, with letters referring to the presented hypotheses.

Based on the theoretical framework and the above hypotheses, the following research model (Figure 1) is developed in this study.

## Materials and methods

### Sample and data collection

In September 2021, data were collected by Norstat Norway through an electronic questionnaire assembled specifically for this research. From Norstat’s panel of 85,000 active participants, there was a total of 1,511 respondents. According to the sociodemographic structure described by Statistics Norway (Statistics Norway, 2022), the sample is considered representative of the Norwegian working population.

The respondents were granted anonymity through a two-step procedure. Norstat had access to their identities for future follow-up studies, but no identity information was shared with the researchers. Further, the respondents were informed about the purpose of the study, about their right to withdraw at any time, and that the data would be used for research only. Any questions that might arise were to be directed to the project leader.

Norstat operates within the Directive 95/46/EC General Data Protection Regulation and complies with Norwegian data protection laws and the main research standards and guidelines described in ICC/ESOMAR and the Quality Management System ISO9001:2015. The Norwegian Centre for Research Data (NSD) approved the research plan and had no comments to the ethical aspects of the research project. At the end of the process, an anonymized complete data file was made available to the research group.

### Measures

#### Remote work

Two items, each with a five-point scale (1 = less than before, 5 = much more than before), were used to measure remote work (Grødem, 2020). One item measured how the COVID-19 restrictions resulted in more remote work, while the other measured whether COVID-19 restrictions resulted in using more digital tools than before the pandemic. Cronbach’s alpha was 0.75.

#### Bullying

Exposure to bullying was measured with 11 items using a trimmed version of the Negative Acts Questionnaire-Revised (NAQ-R) instrument (Einarsen et al., 2009). All items are formulated consistently, avoiding references to the term “bullying” and covering both direct and indirect behaviors. This method may be perceived as more accurate since it does not rely on the respondent’s understanding of bullying (Nielsen et al., 2010). The items assess exposure to negative acts on a five-point scale (1 = Never, 5 = Daily). Cronbach’s alpha was 0.92.

#### Loneliness

Two items developed by Hughes et al. (2004) were used to measure loneliness. The items use a five-point scale (1 = Never, 5 = Daily). One item measured the lack of contact with other people and the other measured the feeling of isolation. Cronbach’s alpha was 0.86.

#### Work engagement

The ultra-short UWES–3 instrument (Schaufeli et al., 2019) was used to measure work engagement. The three items use a five-point scale (1 = Strongly disagree, 5 = Strongly agree) to assess the respondent’s energy, enthusiasm, and immersion at work. Each item represents one aspect of work engagement (vigor, dedication, or absorption). Cronbach’s alpha was 0.81.

#### Control variables

Age and gender were included as control variables in the structural equation model and the correlation matrix.

#### Data analysis

Descriptive statistics, correlations, and Cronbach’s alphas were analyzed using SPSS 26.0, while confirmatory factor analysis (CFA) and structural equation modeling (SEM) were conducted in AMOS 26.0. CFA using maximum likelihood estimation (MLE) was performed to test the validity of the constructs. The measurement model was validated before estimating the structural model (McDonald and Ho, 2002). To analyze the relationships between the latent variables in the developed theoretical model, SEM with MLE was performed. The direction and significance of the beta coefficients potentially support or reject the theoretical model and the associated hypotheses.

Guidelines from (Hu and Bentler, 1999) were used to establish cut-off criteria for the validity and reliability of concepts. The reliability of the concepts is investigated with composite reliability (CR > 0.7) and Cronbach’s alpha (> 0.7). Convergent validity is investigated with average variance explained (AVE > 0.5).

The following indicators and thresholds were used to evaluate the model fit: the comparative fit index (CFI), incremental fit index (IFI), root mean square error of approximation (RMSEA), and standardized root mean squared residual (SRMR). An RMSEA of less than 0.05 indicates a “good” fit, while an RMSEA of less than 0.08 indicates an “acceptable” fit (McDonald and Ho, 2002). For SRMR, a range of 0 to 0.08 is considered “acceptable” (Hu and Bentler, 1999), while for other indicators, values of 0.90 or greater indicate a “good” fit (Hoyle, 1995; McDonald and Ho, 2002). Chi-square was not used to evaluate the model fit due to the large sample size (Bentler and Bonett, 1980; Schumacker and Lomax, 2004).

Bootstrapping was used to test for indirect effects and the mediating role of bullying. Bootstrapping is a method that involves repeatedly sampling from the dataset and estimating the indirect effect in each resampled dataset (Preacher and Hayes, 2008). This method is used before the Sobel test to address indirect effects, as it has high statistical power while also maintaining reasonable control over the Type I error rate (Preacher and Hayes, 2008). Following Hayes’ (2013) recommendations, the data were resampled 5,000 times, and 95 percent bias-corrected confidence intervals (CIs) were estimated.

## Results

### Sample

A total of 1,511 Norwegian workers participated in the study. Among them, 688 were female (45.5%), 771 were between 40 and 66 years old (50.9%), and 602 were less than 40 years old (39.8%). Further, 660 had been in their jobs for 5–20 years (43.7%), while 620 had been in their current jobs for four or fewer years (41%). Of the respondents, 1,053 worked from 21 to 40 h per week (69.7%), and 1,262 were full-time employees (83.5%). The demographic data are presented in Table 1.

**Table 1:** Characteristics of the study participants.

Demographic variables		Total Sample (N = 1,511)	
		*n*	*%*
Gender	Female	688	45.5
	Male	823	54.5
Age	20–24	114	7.5
	25–39	488	32.3
	40–54	486	32.2
	55–66	285	18.9
	67–74	138	9.1
Years in current job			
	≤ 4	620	41
	5–10	379	25.1
	11–20	281	18.6
	≥ 21	231	15.3
Working hours per week			
	≤ 20	119	7.9
	21–40	1,053	69.7
	41–60	308	20.4
	≥ 61	31	2.1
Work situation			
	Full-time	1,262	83.5
	Part-time	243	16.1
	Laid-off	6	0.4

### Descriptive statistics

Descriptive statistics and correlations are presented in Table 2. Participants’ ages varied from 20 to 75 (mean = 45.75, SD = 13.88). Gender was measured on a scale from 0 to 1, where 0 = male and 1 = female (mean = 0.46, SD = 0.50). Excluding control variables, remote work had the highest score (mean = 3.98, SD = 0.87), followed by work engagement with the second highest (mean = 3.38, SD = 0.87). Bullying had the lowest score (mean = 1.27, SD = 0.47), followed by loneliness with the second lowest (mean = 1.57, SD = 0.87). The statistical variation of the different indicators was considered satisfactory.

**Table 2:** Descriptive statistics and correlations.

	Range	Mean	SD	1	2	3	4	5	6
1. Age	20–75	45.75	13.88	–					
2. Gender	0–1	0.46	0.50	−0.10^**^	–				
3. Remote work	1–5	3.93	0.87	0.07^**^	−0.04	–			
4. Bullying	1–5	1.27	0.47	−0.15^**^	−0.02	−0.22^**^	–		
5. Loneliness	1–5	1.57	0.87	−0.11^**^	0.05	0.30^**^	0.57^**^	–	
6. Work engagement	1–5	3.38	0.87	0.14^**^	−0.01	0.12^**^	−0.22^**^	−0.21^**^	–

Gender: 0 = Male, 1 = Female.

** *p* < 0.01.

Relations among measurement concepts were measured by Pearson’s r. The correlations ranged from −0.22 to 0.57. Overall, nine correlations were negative and six were positive. Remote work was negatively correlated to gender and bullying. Bullying was negatively correlated to age, gender, remote work, and work engagement. Further, loneliness was negatively correlated with age, and work engagement, while work engagement was negatively correlated to bullying and loneliness. In general, all correlations were significant (*p* < 0.01), with the exception of correlations with gender.

### Confirmatory factor analysis, reliability, and validity

Confirmatory factor analysis (CFA) was performed using maximum likelihood estimation (MLE) to assess the validity of all the concepts. All dimensions of associated items were included in the assessments (Table 3). CFA supported the measurement model with a “good” fit (CFI = 0.91, IFI = 0.91, RMSEA = 0.07, SRMR = 0.05). The standardized factor loadings ranged from 0.63 to 0.98. Bullying had the lowest loading (0.63) with “being ignored or excluded,” while loneliness had the highest loading (0.98) with “How often do you feel isolated from others?” Moreover, CR was above 0.7, AVE was above 0.5, and Cronbach’s alphas ranged from 0.75 to 0.92, with remote work being the lowest (0.75) and bullying being the highest (0.92). Based on the overall results and the model fit, the factor-to-item relations were considered satisfactory. Therefore, the structural model could be tested with a validated measurement model.

**Table 3:** Confirmatory factor loadings with standardized factor loadings, reliability, and convergent validity.

**Dimension/Item**	**Factor loadings**	**CR**	**AVE**	**Alpha**
*Remote work*		0.76	0.61	0.75
Have the measures led to you working from home?	0.80			
Have the Covid-19 restrictions led to you using digital tools more often than before (Skype, Teams, Zoom, and similar services)?	0.76			
*Bullying*		0.92	0.52	0.92
Being ignored or excluded.	0.63			
Having insulting or offensive remarks made about your person, attitudes, or your private life.	0.74			
Being shouted at or being the target of spontaneous anger.	0.65			
Repeated reminders of your errors and mistakes.	0.74			
Being ignored or facing a hostile reaction when you approach.	0.76			
Persistent criticism of your errors or mistakes.	0.79			
Having your opinion ignored.	0.76			
Practical jokes carried out by people you do not get along with.	0.75			
Being the subject of excessive teasing and sarcasm.	0.67			
Someone withholding information which affects your performance.	0.68			
Spreading of gossip and rumors about you.	0.72			
*Loneliness*		0.87	0.78	0.86
First, how often do you feel that you lack companionship?	0.77			
How often do you feel isolated from others?	0.98			
*Work engagement*		0.82	0.60	0.81
At my work, I feel bursting with energy.	0.75			
I am enthusiastic about my work.	0.90			
I am immersed in my work.	0.66			

CR = composite reliability; AVE = average variance explained; Alpha = Cronbach’s alpha.

### Result of structural equation modeling

**Figure 2:**
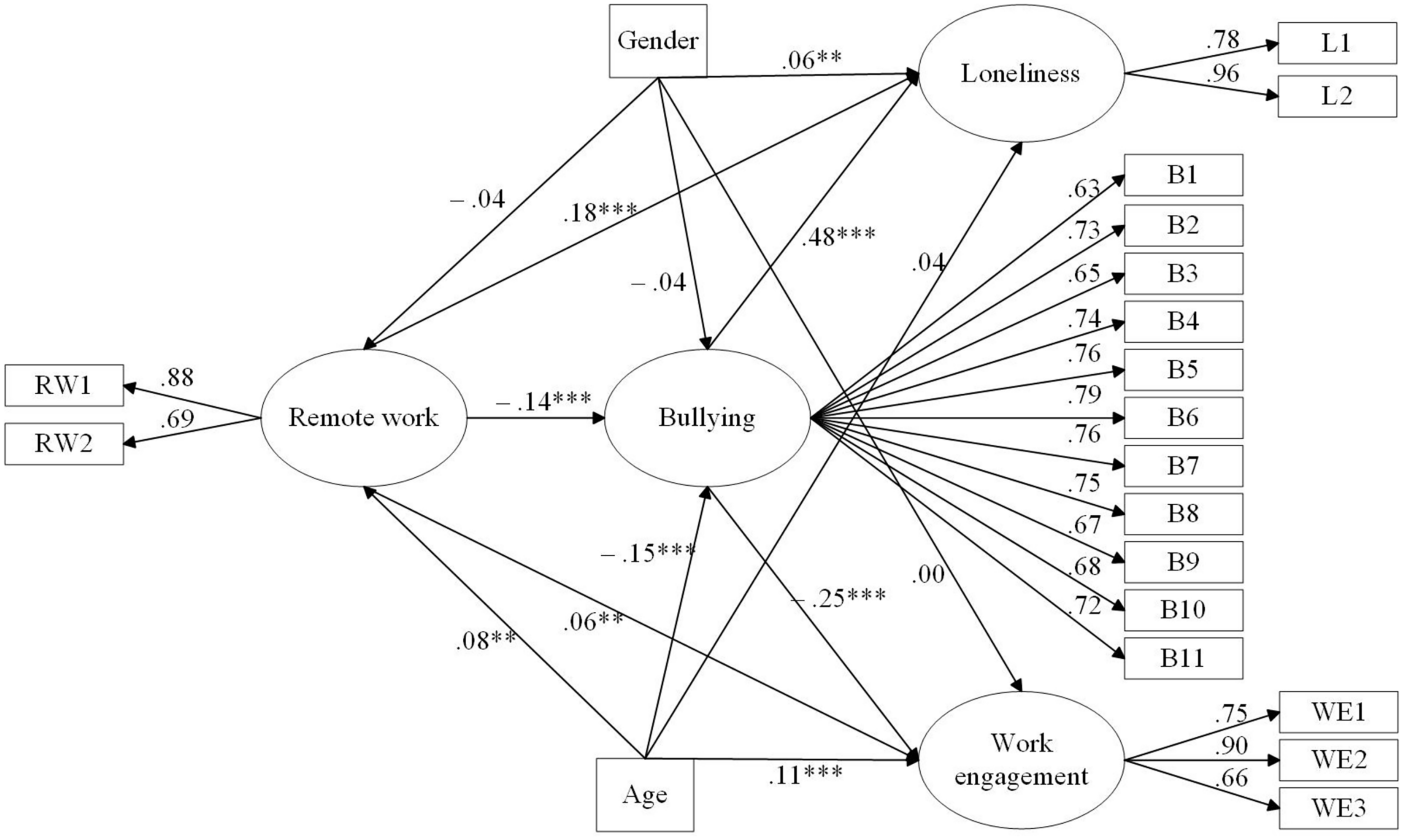
Result of structural equation modeling conducted on Norwegian workers with standardized path coefficients. Gender (0 = Male, 1 = Female); ^**^ p < 0.05, ^***^ *p* < 0.01.

The theorized model (Figure 2) with the control variables applied was tested using SEM and maximum-likelihood extraction. All model fit indicators were above the recommended thresholds (CFI = 0.91, IFI = 0.91, RMSEA = 0.07, SRMR = 0.05); thus, the model fit of the structural model was considered “acceptable..” All the beta coefficients were significant and consistent with the hypothesized directions, they are presented in Table 4. Remote work was positively related to loneliness (*β* = 0.18, *p* < 0.01), supporting hypothesis 1. Remote work was positively related to work engagement (*β* = 0.06, *p* < 0.05), supporting hypothesis 2. Additionally, remote work was negatively related to bullying (*β* = −0.14, *p* < 0.01), supporting hypothesis 3. Moreover, bullying was positively related to loneliness (*β* = 0.48, *p* < 0.01) and negatively related to work engagement (*β* = −0.25, *p* < 0.01), supporting hypotheses 4 and 5. In total, the model explained 5% of the variance related to bullying, 25% of the variance related to loneliness, and 9% of the variance related to work engagement.

**Table 4:** Standardized path coefficients (direct effects).

**Hypotheses**	**Relationships**	**β**	**p**
H1	Remote work→Loneliness	0.18	0.001
H2	Remote work→Work engagement	0.06	0.048
H3	Remote work→Bullying	−0.14	0.001
H4	Bullying→Loneliness	0.48	0.001
H5	Bullying→Work engagement	−0.25	0.001

Regarding the control variables, age had three significant relations while gender had one. Age was positively related to remote work (*β* = 0.08, *p* < 0.05), negatively related to bullying (*β* = −0.15, *p* < 0.01), and positively related to work engagement (*β* = 0.11, *p* < 0.01). Gender was positively related to loneliness (*β* = 0.06, *p* < 0.05), indicating that men were lonelier than women.

### Mediation by bullying

Bootstrapping was used to test for indirect effects. With the data resampled 5,000 times, two significant indirect effects were discovered, these are presented in Table 5. (H6a) remote work → bullying → loneliness (standardized indirect effect = −0.07, *p* < 0.001; 95% CI = −0.11, −0.04), and (H6b) remote work → bullying → work engagement (standardized indirect effect = 0.04, *p* < 0.001; 95% CI = 0.02, 0.06). Hence, the results support hypotheses 6a and 6b since bullying mediates remote work’s influence on loneliness and work engagement.

**Table 5:** Specific indirect effects.

**Hypotheses**	**Relationships**	**β**	**p**
H6a	Remote work→Bullying→Loneliness	−0.07	0.001
H6b	Remote work→Bullying→Work engagement	0.04	0.001

## Discussion

Research on remote work and social distancing has accelerated in the aftermath of COVID-19. However, this is the first study exploring the relationships between remote work, loneliness, work engagement, and bullying through a theoretical model that includes all factors simultaneously. The study was conducted almost two years into the COVID-19 pandemic with a large representative sample of Norwegian workers. Remote work seems to protect against bullying for workers in Norway. This finding is both interesting and important since bullying is associated with multiple unwanted outcomes (Bartlett and Bartlett, 2011; Nielsen and Einarsen, 2012; Branch et al., 2013). This study builds on previous research indicating the destructive mechanisms related to bullying work behaviors. The findings confirmed the hypotheses that bullying is negatively related to work engagement and positively related to loneliness. The findings indicate that bullying partially mediates remote work’s influence on loneliness and engagement. Further, remote work is positively related to both loneliness and work engagement. Hence, these findings show that remote work leads to both negative and positive outcomes.

### Remote work and loneliness

Our study provides evidence of a positive association between remote work, loneliness, and work engagement, supporting H1 and H2. These results are also supported by previous studies that highlight changes in social interactions with colleagues, exposure to social isolation, and employee engagement when working remotely (e.g., Hwang et al., 2020; Ozimek, 2020; Palumbo, 2020; Spurk and Straub, 2020; Buecker and Horstmann, 2022). The positive relation of remote work and loneliness may be explained by the increased difficulty in maintaining interpersonal relationships, which is an important element of counteracting loneliness. Further, this positive association may be linked to the forced isolation in everyday life caused by governmental restrictions. According to Szkody et al. (2021), people living with family or friends during the pandemic could also experience heightened feelings of loneliness as a result of being cut off from other previously available resources. However, according to Heidinger and Richter (2020), people living alone reported higher levels of loneliness than those in multi-person households. Nevertheless, there was no significant difference in the perceived loneliness of those living alone before and during COVID-19. This suggests that loneliness during the pandemic could be related to isolation from life as we know it rather than simply being linked to the loss of interpersonal relationships.

### Remote work and work engagement

Previous research suggests that people experiencing loneliness have lower levels of work engagement (Jung et al., 2021). Therefore, an interesting finding in our study is a dualism of remote work, which is positively related to loneliness and simultaneously, positively related to work engagement. Remote work offers more flexibility and autonomy, both of which have been shown to increase work-life balance and work engagement (Eek and Axmon, 2013; De Spiegelaere et al., 2016). Furthermore, the change in workplace removes work-related interruptions (e.g., questions from colleagues and informal discussions) and commuting time. Commuting can be stressful (Beňo, 2021), and extensive commuting has been shown to negatively affect mental health (Hilbrecht et al., 2014) and work engagement (Gerpott, 2021). In this sense, remote work can be both positively related to work engagement and negatively related to loneliness. However, Szkody et al. (2021) found that people with high levels of social support before the lockdown felt more lonely during isolation as they could no longer physically access their existing social networks. Further, levels of perceived loneliness were particularly high during the pandemic (Killgore et al., 2020). Hence, these previous studies somewhat support the findings of this study. These findings call for more research on perceived loneliness in cases of remote work. The dynamics and outcomes of remote work might change after the pandemic when employees return to their normal social lives and remote work is no longer compulsory. It will be much easier for remote workers to connect socially when the pandemic is over, at which point the negative relation of remote work to loneliness might diminish.

### Remote work and bullying

Our study indicates that remote work functions as a protective mechanism against bullying, which is a very interesting finding that supports hypothesis H3. Bullying should be taken seriously as it is considered as one of the most detrimental stressors in working life (Björklund et al., 2019).

Remote work potentially involves fewer social interactions. Fewer social interactions might protect against or reduce bullying. This is supported by a study by Bacher-Hicks et al. (2022), who found a dramatic decrease in bullying during the pandemic due to fewer in-person interactions. Another study found that people who already experienced low social support benefitted from isolation (Foulkes and Blakemore, 2021; Szkody et al., 2021). In these cases, isolation can improve psychological health since it removes reminders of one’s low level of social support (e.g., one no longer witnesses social lunches in the cafeteria, small talk in the hallway, etc.; Szkody et al., 2021). Scholars have also investigated the potential spillover effect where workplace bullying is transferred into cyberbullying (Stich, 2020; Ezerins and Ludwig, 2021). However, a study by Bacher-Hicks et al. (2022) found cyberbullying to decrease during the pandemic, proving that cyberbullying is strongly related to in-person bullying.

Building on this research, it can be argued that organizations can use remote work as a measure to address or reduce bullying. However, remote work must be considered carefully, as it does not solve the underlying issues of bullying and could potentially escalate relational problems if not handled properly (Keashly et al., 2011).

### The mediating role of bullying

In line with other studies in the field (Ireland and Power, 2004; Rai and Agarwal, 2017; Einarsen et al., 2018; Bai, 2021), bullying was found to be positively related to loneliness and negatively related to work engagement, supporting H4 and H5. These results add to existing empirical research documenting the unwanted negative outcomes of bullying. These negative outcomes were also found in this study, conducted during the final stage of pandemic lockdown among Norwegian workers.

This study also investigated the mediating role of bullying. Interestingly, the results revealed that bullying partially mediates the influence of remote work on loneliness and work engagement, supporting H6a and H6b: bullying suppresses the positive influence of remote work on loneliness and strengthens the positive relationship between remote work and work engagement. This suggests that when victims of bullying, work remotely, they are more likely to experience lower levels of loneliness and be more engaged in their work, thus making social restrictions a welcome relief for bullied victims (Foulkes and Blakemore, 2021). Previous research indicating that in-person interactions are positively associated with bullying (Bacher-Hicks et al., 2022; McFayden et al., 2021) supports this finding. Although speculative, some of the effect may be explained by the perception of increased autonomy when working remotely (Bosua et al., 2017; Schall, 2019). Higher autonomy is positively related to work engagement (Bošković, 2021; Galanti et al., 2021) and negatively associated with bullying (Bowling and Beehr, 2006; Balducci et al., 2011; Rousseau et al., 2014) and loneliness (Henning et al., 2021; Wang et al., 2021). This finding could suggest that when bullied victims work remotely, they experience fewer in-person interactions and higher autonomy, both of which are expected to have desired effects on work engagement, bullying, and loneliness.

## Theoretical and practical implications

This study contributes to the literature on remote work by proposing a theoretical model including bullying, loneliness, and work engagement. Our findings offer valuable implications into the detrimental mechanisms related to in-person interactions for victims of workplace bullying. Furthermore, this study implicates that in-person interactions are major contributors to workplace bullying; thus, remote work and the associated perception of higher autonomy might prevent workplace bullying. Hence, implying that remote work could be considered when employees have high levels of sensitivity to the work environment, and managers could consider using this tool in periods with high levels of harassment or conflict. Based on the enormous increase in remote work during COVID-19 and the associated up- and downsides, it is important to interpret the findings in situations when the workforce returns to the workplace free of COVID-19 restrictions. Some organizations and employees may not want to return to the ways they operated before, as remote work’s value has been recognized and accepted (Savić, 2020). Furthermore, many managerial tasks and HR strategies could potentially be redefined by the situation caused by the pandemic.

The results can be applied to design work arrangements with the individual—not solely the organization—in mind to present risk of bullying. Top management cannot simply implement remote work as a common standard, as individuals may need different arrangements (Gratton, 2021) due to personality differences (Bai, 2021). Therefore, work design may be a concern for local managers as they work more closely with employees. Remote work affects employees both positively and negatively. Thus, organizations should try to optimize the benefits and understand the trade-offs. As our findings indicate, during COVID-19, employees felt lonelier when working remotely. Organizations should therefore implement measures to prevent this increase in loneliness. One such measure could be “hybrid work,” working from home one or two days per week. Hybrid work allows employees to maintain interpersonal relationships and regular contact with co-workers while reaping the benefits of remote work, ultimately decreasing loneliness while maintaining high levels of work engagement. However, it is important that the arrangements do not create unfairness between employees (Gratton, 2021). Moreover, this study recommends that organizations implement a remote work policy as a measure against bullying. The theoretical implications of this study indicate that bullied victims benefit the most from working remotely. By separating the bully from the target, exposure to negative acts is reduced and remote work may act as a temporary solution until the underlying issue is addressed. These theoretical implications should be further developed in forthcoming studies.

## Limitations and future research

Our current study has many strengths: it was based on a representative sample of workers in Norway during the pandemic lockdown. However, some limitations must be acknowledged. The study uses a cross-sectional design, meaning that it is unable to determine the causation or direction of the effects. The pandemic brought extensive restrictions to society, interfering with our social, professional, and personal lives. This could make the participants more prone to other factors that potentially lead to loneliness. Furthermore, as the data are self-reported, the results may have been influenced due to common method variance. However, several measures such as CFA, AVE, and CR were applied to control the validity and objectivity of the study. Moreover, as our aim for this study was to investigate how remote work influences employees, self-reported appraisals are a great tool for identifying the perceptions and reactions of interest (Spector, 1994). The use of such a measure is therefore appropriate. However, a longitudinal study is likely needed to control the findings of the present study. Therefore, a before-and-after study on the impact of remote work over a period of time is an important avenue for further research. This study did not investigate the relation between loneliness and work engagement, further research should consider this relation.

Another important note is that the participants in this study were Norwegian employees during the pandemic. Thus, our findings should be interpreted with some limitations in mind regarding generalization. Similar studies at different locations could help generalize and supplement our findings. Moreover, prior relevant research studies are limited. This presents an issue for this study but also indicates the importance of expanding research to cover the gap in the existing literature.

## Conclusion

In conclusion, this study explores the relationship between remote work, loneliness, work engagement, and bullying among Norwegian workers during COVID-19 restrictions. The results suggest that employees felt lonelier when working remotely but experienced increased engagement in work, illustrating that remote work affects both mental health and productivity. The results also suggest that remote work reduced bullying and played a mediating role in the associations between remote work, loneliness, and work engagement. Remote work does not affect all employee’s equally, bullied victims were found to benefit most from working remotely, indicating a protective function against bullying. Hence, this study finds that remote work is related to both positive and negative mechanisms at work. Since remote work is positively related to both loneliness and work engagement, this study illustrates a distinct advantage of remote work, but its associated issue of loneliness cannot be overlooked. Therefore, it is suggested that organizations should consider more moderate levels of remote work in the aftermath of COVID-19. This study contributes to the established literature of remote work, extending our knowledge of remote work’s long-term impact on employees. Future research may examine differences in the effect of remote work during COVID-19 and after.

## Data availability statement

The raw data supporting the conclusions of this article will be made available by the authors, without undue reservation.

## Author contributions

VB: introduction, article drafting, literature review, implication, limitation, and discussion. J-SA: method, analysis, result, literature review, implication, and conclusion. EO: data collection, supervision, study design, revised manuscript, and assessment support. All authors contributed to the article and approved the submitted version.

## Funding

This study was funded by the UiS Business School, Department of Innovation, Leadership and Marketing.

## Conflict of interest

The authors declare that the research was conducted in the absence of any commercial or financial relationships that could be construed as a potential conflict of interest.

## Publisher’s note

All claims expressed in this article are solely those of the authors and do not necessarily represent those of their affiliated organizations, or those of the publisher, the editors and the reviewers. Any product that may be evaluated in this article, or claim that may be made by its manufacturer, is not guaranteed or endorsed by the publisher.
